# Distribution and Genetic Characterization of Border Disease Virus Circulating in Sardinian Ovine Flocks

**DOI:** 10.3390/pathogens9050360

**Published:** 2020-05-09

**Authors:** Ilaria M. Piras, Silvia Dei Giudici, Manlio Fadda, Antonio G. Anfossi, Annalisa Oggiano, Marco Pittau, Bernardo Chessa

**Affiliations:** 1 Department of Veterinary Medicine, University of Sassari, 07100 Sassari, Italy; ilaria.piras@ucdconnect.ie (I.M.P.); manfadda@uniss.it (M.F.); aanfossi@uniss.it (A.G.A.); pittau@uniss.it (M.P.); 2 Istituto Zooprofilattico Sperimentale della Sardegna, 07100 Sassari, Italy; annalisa.oggiano@izs-sardegna.it

**Keywords:** BDV, epidemiology, genetic characterization, Sardinia

## Abstract

Border Disease (BD) is a worldwide distributed pathology accountable for significant losses in the sheep and goat farming industry. The etiological agent is a *Pestivirus* within the family *Flaviviridae* called *border disease virus* (BDV). Despite the Sardinian ovine population being by far larger than any other Italian region, the prevalence and distribution of BD on the island are unknown. Here, we aim to determine the distribution of BDV in sheep flocks and to genetically characterize the circulating strains in Sardinia. The geographical distribution, antibody positivity, and viral genome presence have been analysed for 1286 sheep flocks distributed all over the island from bulk tank milk sampled between May 2014 and 2015. Of the flocks tested, 11.28% (95% CI 9.66–13.12) resulted positive for the presence of anti-pestivirus antibodies with an uneven distribution between Sardinian provinces. In addition, using RT-PCR, nine BDV genomes were amplified from milk pellets of the seropositive samples. Phylogenetic analysis revealed that all the viruses amplified clustered in the same group classified as BDV-7. This represents the first study on the distribution of pestivirus infection and genetic characterization of BDV strains circulating in the Sardinian sheep population. Future studies are needed to clarify the origin, the evolution, and the epidemiology of BDV-7 in Sardinia.

## 1. Introduction

Border disease virus is (BDV) is the aetiologic agent of border disease (BD) of sheep and goats and is accountable for important economic losses worldwide [1]. BDV is an important agent of in utero infection causing embryonic and foetal death; congenital malformations; and birth of weak, shaky lambs with typical wool abnormalities (hairy shakers) [1]. Less frequently, the intrauterine infection results in clinically normal lambs that can be persistently infected with BDV [2]. Persistently infected (PI) animals are asymptomatic and almost invariably seronegative and shed the virus constantly during the course of their life. Horizontal transmission happens fast within a herd through oral, conjunctival, and intranasal contact with contaminated secretion and excretion of viremic animals [3]. Viremia last a few days in transient BDV infection but is lifelong in PI individuals that are considered the major virus reservoir [2]. When first entering a susceptible flock, BD causes conspicuous losses that normally drop during the following years as adult sheep acquire immunity [3]. The introduction of naïve ewes, though, would maintain the number of losses significant [3].

*Border Disease Virus* (BDV) belongs to the genus *Pestivirus* within the family *Flaviviridae* [1] and is closely related to are *bovine viral diarrhoea virus 1* and *2* (BVDV-1 and BVDV-2) and *classical swine fever virus* (CSFV) [4].

*Pestiviruses* are small single-stranded, positive-sense RNA viruses. Their genome contains two untranslated regions (UTRs) at the 5′ and 3′ ends and an open reading frame (ORF), encoding a polyprotein which is processed into four structural proteins (C, E^rns^, E1, and E2) and 8 nonstructural proteins (N^pro^, p7, NS2, NS3, NS4A, NS4B, NS5A, and NS5B) [1]. For genetic typing, analyses of the 5′ UTR or the N^pro^ gene are most commonly used [5,6,7,8,9,10]. 

BDV genetic diversity is greater than other pestivirus species, and European isolates are phylogenetically segregated into seven genotypes, namely BDV-1 to BDV-7 [5,9,11,12,13,14]. Recent studies, though, suggest the presence of a novel putative border disease virus genotype 8 (BDV-8) [15,16,17]. In Italy, *Pestivirus* infection in sheep and goats have been described in the southern regions since the 1990s [18,19], but no studies so far have been published on the prevalence of *Pestivirus* infection in small ruminants in Sardinia. 

About 3 million sheep and 12,000 farms are involved in the Sardinian dairy industry. All sheep farmed in Sardinia belong to the Sarda breed, therefore accounting for 70% of the national Sarda sheep entity and 40% of the total Italian sheep stock [20]. 

Sarda sheep is an autochthonous Sardinian breed. This small and frugal sheep has been selected to the highest standards over the last 100 years to create one of the most competitive breeds for the production of milk on the global scene [21]. Dairy production is the most traditional and florid industry of the island. The most popular product of Sardinian dairy industry is by far the “pecorino” cheese, which is clotted using ovine milk. Sardinian pecorino cheese exports were calculated over 16.000 tons in 2018, with an increase of 18% in 2020, and the business orbiting around the USA marked alone (which absorbs approximately 50% of pecorino cheese exports) is calculated around 100 million dollars [22]. Considering these numbers, the economic impact of BD on small ruminant productions might be underestimated on the island. This is the first study to determine the distribution of pestivirus infection in Sardinian sheep flocks and to genetically characterize the strains circulating in the island.

## 2. Results

### 2.1. Distribution of Pestivirus in Ovine Flocks in Sardinia

**Figure 1:**
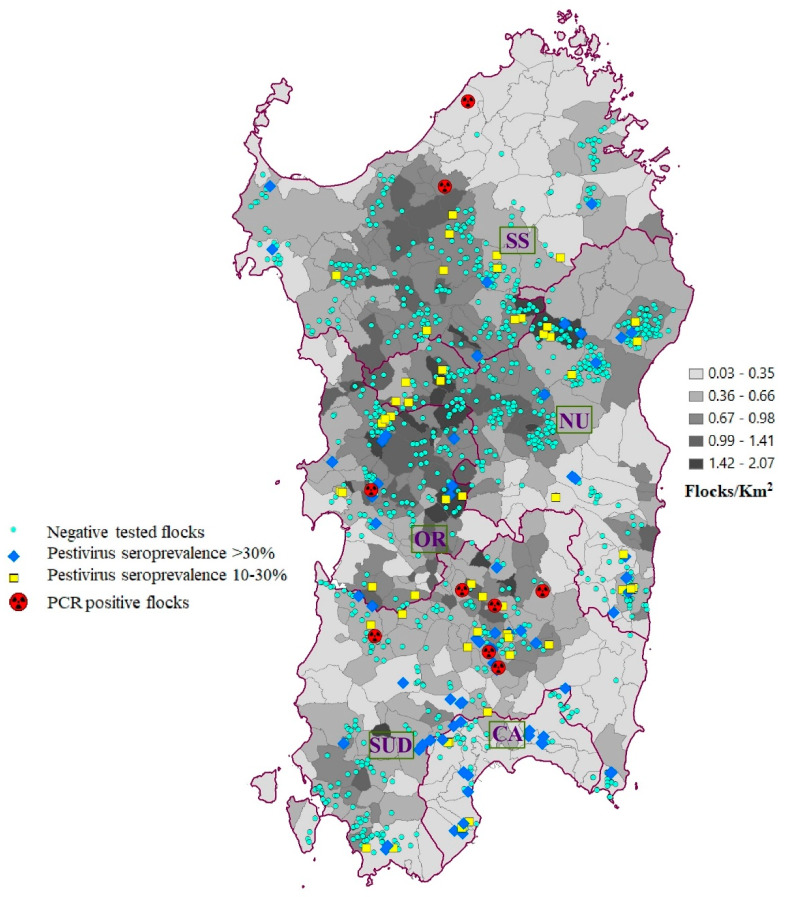
Sheep density, location of sampled sheep flocks, and distribution of flocks positive in antibody ELISA and flocks positive in RT-PCR.

*Pestivirus* antibodies were detected in 145 bulk tank milk samples (11.28%) out of 1286 sheep flocks included in this study. Geographic Information System (GIS) analysis of the geographic distribution of *Pestivirus*-positive samples is represented in Figure 1. The flock seroprevalence was highly variable among provinces, as shown in Table 1 and represented in Figure 1. The highest seroprevalence was observed in the province of Cagliari (CA), with 37.5% of positive flocks, and in Sud Sardegna (SU), with 16.35% positive flocks, whereas in the provinces of Sassari (SS) and Nuoro (NU), the lowest percentages were recorded with 5.84% and 7.44% of positive flocks respectively. In the province of Oristano (OR), specific antibodies were detected in 11.11% of the flocks tested. As revealed by the overlapping confidence intervals, some differences in flock seroprevalence were not statistically significant [23]. No differences were observed between the Sassari, Nuoro, and Oristano provinces; instead, Sassari and Nuoro flock seroprevalences were significantly lower than those of Cagliari and Sud Sardegna. Significative differences were also found between Oristano and Cagliari and between Sud Sardegna and Cagliari provinces. Further, based on bulk tank milk antibody inhibition percentage (AIP) value, within-flock seroprevalence was estimated. Considering this estimate, the proportion between flocks with higher within-flock seroprevalence (over 30%) and lower within-flock seroprevalence (between 10–30%) is approximately 1 to 1 in the majority of provinces (Table 1). In the province of SU, the herds with higher within-flock seroprevalence are more than twice the number of flocks with lower within-flock seroprevalence, and in the CA province, the proportion between the same flocks reaches a 3-to-1 ratio (Table 1).

### 2.2. Genetic Characterization of the Circulating Strains in the Island.

**Figure 2:**
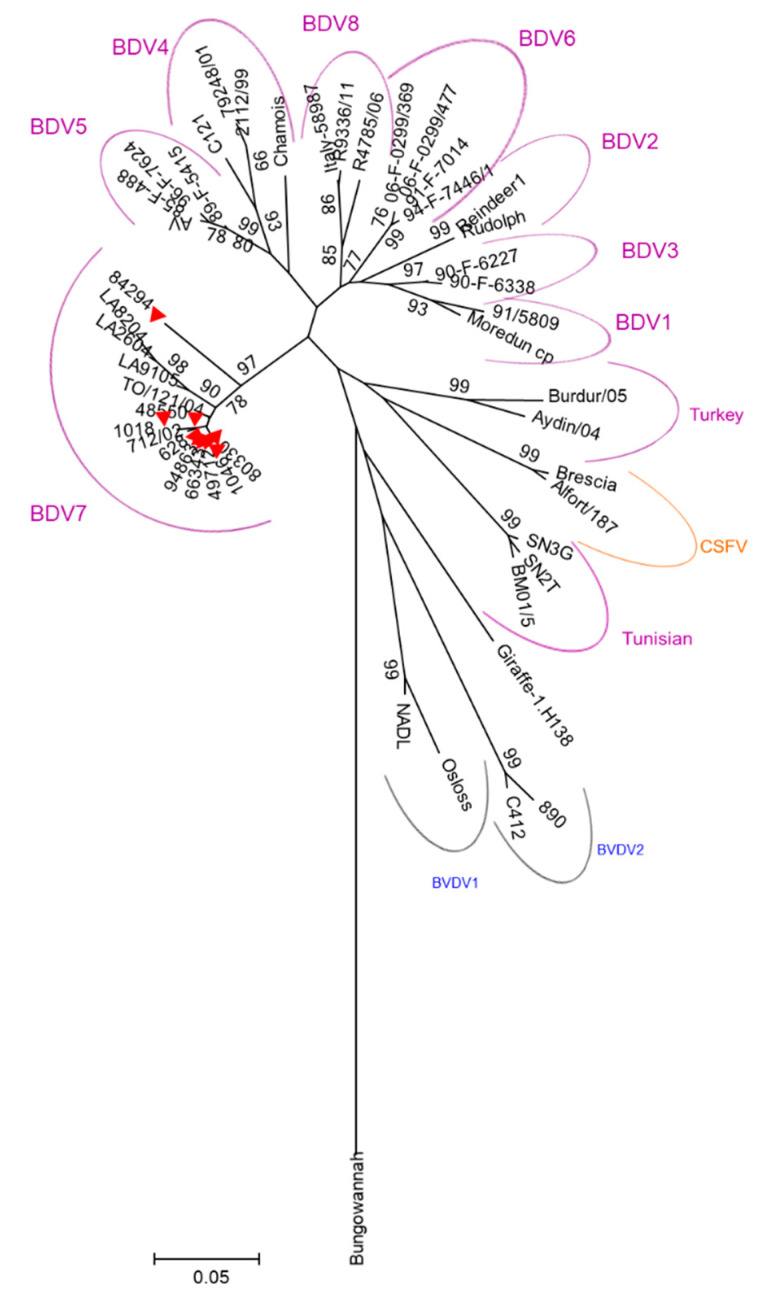
Phylogenetic analysis of the strains sequenced in this study: The phylogenetic tree is based on 244 bp sequence amplified from the the 5’ UTR region of Sardinian *Pestivirus* strains (red triangles) and other 40 reference *Pestivirus* strains sourced from GenBank. Neighbor-joining algorithm with the Kimura 2-parameter method was used to calculate the evolutionary history. A bootstrap test was used to calculate the percentage of replicate trees (1000) in which the associated taxa clustered together [24]. The tree was grouped out the *Bungowannah pestivirus* sequence (DQ901402). MEGA5 was the tool for evolutionary analyses. The bar indicates the number of substitutions per site.

The pellet obtained from ELISA positive milk samples were further tested by RT-PCR for virus genome detection. From 145 positive samples, 9 were found positive after amplification of a product of the expected size of 244 bp. The characteristics of the strains analyzed are reported in Table 2. Sequencing data indicates that all samples amplified match BDV genomes. The sequences amplified in this study are available in GenBank under the accession numbers MH733598 and MH733606 (Table 2). In addition, phylogenetic analysis based on the 5’ UTR revealed that all the 9 Sardinian strains clustered in the same BDV-7 group (Figure 2) containing the only five sequences reported so far from strains isolated in Italy.

Sardinian BDV strains showed 92.5–100% similarity between them and 90.5–99% with the other Italian sequences. 

Figure 1 represents municipalities, limits, and names of the provinces and the density of sheep in Sardinia, where the yellow square is pestivirus within-flock seroprevalence between 10–30%, the blue rhombus is pestivirus flock seroprevalence > 30%, the red circle is PCR positive flocks, and the green circle is negative flocks.

## 3. Discussion

The present study aims to determine the seroprevalence and distribution of *Pestiviruses* in Sardinian sheep flocks using ELISA screening on skimmed bulk tank milk. The ELISA kit adopted in this study was developed by IDEXX for bovine serum, plasma, and milk and ovine serum. Berriatua et al. [25], Corbière et al. [26], and García-Pérez et al. [27] validated the kit for ovine milk and bulk tank milk, demonstrating 100% agreement with criteria for antibody status and estimated within-flock seroprevalence [25]. 

Our results indicate a Sardinian flock seroprevalence of 11.28% (95% CI 9.66–13.12). This value is considerably lower compared to neighboring countries like Austria that reports 73.9% [28] and Switzerland that reports values between 30.1% and 67% [29,30] or other Mediterranean countries like Algeria [31] that reported 98% of *Pestivirus* flock positivity and Morocco with 93% [32]. Also, flock seroprevalence in the Spanish Basque country is considerably higher than Sardinia with average values of 68% [25]. This value, though, might be underestimated as the ELISA kit used fails to identify flocks with less than 10% seropositive lactating animals. 

Interestingly, the distribution of the flock seroprevalence was uneven between Sardinian provinces, with the territory of Cagliari hosting more than 37% ELISA positive flocks. Further, considering the estimated within-flock seroprevalence, the Cagliari territory also stands out among other provinces, showing an increased proportion of flocks with > 30% positive lactating ewes within the flock (Table 1). These results, based on estimated AIP values from bulk tank milk, suggest the presence of recent virus exposure in the flocks within this province most likely due to an increased presence of PI animals among breeders. In fact, even if PI animals are considered more susceptible to secondary disease, often, they appear clinically normal and survive to sexual maturity [1]. As PI individuals represent a constant source of infectious virus, their identification becomes essential in any control program. All traded sheep should be screened for the presence of viral RNA or antigens (using antigen ELISA) associated to BDV antibody negativity. 

Finally, the circulating strains in Sardinia were genetically characterized. Genetic classification is important to enhance knowledge of BDV epidemiology. In the past, few studies analyzed pestivirus strains circulating in small ruminants in Italy. Giammarioli et al. [9] analyzed five small ruminant isolates collected from 2002 to 2005 in central Italy and revealed that they clustered in the novel phylogenetic group BDV-7. In 2011, BD was reported also in goat herds in northern Italy and the etiological agent was identified as BDV-3 [33]. In 2015, the genetic heterogeneity of small ruminant isolates in the country was further investigated; Italian isolates collected from 2002 to 2014 from central (Toscana, Lazio, Marche) and southern (Basilicata) regions were classified in four distinct genetic groups: BDV-1, BDV-3, BDV-5, and BDV-7 [34]. The novel putative BDV-8 was first described in Switzerland in 2010 [17] and recently described in a goat and in a chamois in northwestern Italy [15,16]. A recent study reported the presence of BVDV-1 and Tunisian-like *Pestivirus* in small ruminant in Sicily [35]. 

To date, BDV-7 has been reported in Italy alone and specifically in Toscana and Lazio regions [9,34]. Interestingly, these regions have the highest number of Sarda sheep in the peninsula [20] as they represent the very first regions where Sarda sheep were exported following the emigration of Sardinian farmers in central Italy during the 1960s [21]. Official data from AssoNaPa [36] recognizes the trade direction of Sarda sheep to be exclusively outside Sardinia as the island detains 99% of the animals registered to the official genealogic book. These genetic “elite” flocks were 15,000 farms all over Italy sourced for Sarda sheep [36]. Our results showed that BDV-7 was the only genotype isolated in Sardinian sheep. Considering the size of the Sardinian ovine population; the levels of antibody prevalence in the southern region of the island, higher than any other region in Italy; and the prevalent directions of livestock trade, we consider the possibility that the new genotype BDV-7 first originated in Sardinia and then spread to other Italian regions along with the movement of infected sheep towards the Italian peninsula. Other future analyses such as Bayesian and phylogeographic reconstruction could allow us to define the number of BDV introductions on the island and to confirm our hypothesis about the direction of its spreading. The full genome sequence of Sardinian BDV-7 strains and its comparison with other Italian or European BDV strains would provide more information to understand BDV epidemiology. Future studies should be led in order to understand the origin, the evolutionary history, and the epidemiology of BDV-7 in Italy.

## 4. Materials and Methods 

### 4.1. Selection of Sheep Flocks 

Samples were collected from 1286 sheep flocks in Sardinia in 2014 and 2015. Due to the absence of previous prevalence studies performed in Italy, the sample size was calculated using an expected proportion of 0.5, a confidence level of 95%, and a confidence interval of 2.6%. As we tested the samples, we realized there was a significantly higher flock seroprevalence in Cagliari and, subsequently, we decided to expand the simple size from this area to confirm the preliminary results. The sampling in this province was expanded from 10% to 25%. Province, number of flocks, and ewes of each flock analyzed are reported in Table 1. The number of sampled sheep farms corresponded to 11.15% of all registered farms in Sardinia, and the sampling was performed randomly stratifying for provinces (Figure 1). 

### 4.2. Analysis of Samples and Collection of Data

Bulk tank milk (BTM) was collected randomly from farms and transported in cool boxes to the laboratory (Department of Veterinary Medicine, University of Sassari), where they were tested for pestivirus antibodies and for virus, as described below. 

Samples (20 mL of milk) were centrifuged for 15 min at 3500× *g* at 4 °C and de-fatted, and for each sample, 1 mL of skimmed milk was transferred to 1.5 mL microtubes and frozen at −80 °C until analyzed (serological analysis). Supernatant was subsequently discarded; cellular pellets were washed twice with Phosphate Buffered Saline (PBS) and resuspended in 0.2 mL of PBS and stored at −80 °C until analyzed (virological analysis). As a negative control, 0.2 mL of PBS was collected and stored at −80 °C till analysis. 

### 4.3. Serological Testing 

Presence of pestivirus antibody in bulk tank milk was determined by ELISA, using a “BVDV/MD/BDV p80 Protein Antibody Test Kit” (IDEXX, Hoofddorp The Netherlands); following manufacturer’s instructions, AIP was calculated as AIP% = Optical Density (OD) 450 nm of the sample(S)/OD 450 nm of the negative control (N) × 100. Within-flock seroprevalence was estimated according to AIP (antibody inhibition percentage) values as described for bovine BTM samples. In detail, AIP values equal or below 45% estimate a seroprevalence above 30% in the lactating animals within the flock; values between 45% and 80% estimate a seroprevalence between 10–30% of the same animals.

### 4.4. Virological Testing 

Viral RNA was extracted using the QIAamp MinElute Virus Spin kit (QIAGEN, Hilden, Germany) from the cellular fraction of each ELISA pestivirus positive sample, according to manufacturer’s instructions. RNA was retro-transcribed using the GoScript™ Reverse Transcription kit (PROMEGA, Madison, USA), according to manufacturer’s protocol. Then a 5-μL aliquot of cDNA was used as template for PCR amplification with Taq DNA polymerase (QIAGEN, Hilden, Germany) kit. The panpestivirus primers 324 (ATGCCCt/aTAGTAGGACTAGCA) and 326 (TCAACTCCATGTGCCATGTAC) [37] were used, and the following thermal profile was adopted: 94 °C for 3 min; 35 × (94 °C for 60 s; 55 °C for 60 s; and 72 °C for 30 s); 72 °C for 10 min; and a 4 °C hold. 

### 4.5. Sequencing and Phylogenetic Analysis 

Amplicons were separated using 2% agarose gel and were subsequently purified using QIAquick Gel Extraction Kit (QIAGEN, Hilden, Germany), accordingly to manufacturer’s instruction. Purified samples were stored at −20 °C until analysis.

Sequencing was performed using the primers mentioned before on an ABI-PRISM 3500 Genetic Analyzer (Applied Biosystems, Foster City, CA, USA) with a DNA sequencing kit (dRhodamine Terminator Cycle Sequencing Ready Reaction; Applied Biosystems, Foster City, CA, USA). The consensus sequences were assembled and edited in the BioEdit software, version 7.0.0 [38]. 

Phylogenetic tree was constructed using 244 nt from the 5’ UTR region of the pestivirus sequences found in this study and of 40 reference strains retrieved from GenBank: BDV-1: Moredun/cp (U65022.1), 91/5809 (AF026768.1), X818 (AF037405.1), and BD31 (U70263.1); BDV-2: Rudolph (AB122086.1), Reindeer-1 (AF144618.2), and Chemnitz (EU637066.1); BDV-3: 90-F-6338 (EF693991.1), 90-F-6227 (EF693989.1), Gifhorn (KF925348.1), and FNK2012-1 (KC963426.1); BDV-4: C121 (DQ275625.1), 2112/99 (AY159513.1), 79248/01 (AY159515.1), and H2121 (GU270877.1); BDV-5: Aveyron (KF918753.1), 89-F-5415 (EF693988.1), 85-F-488 (EF693985.1), and 96-F-7624 (EF693998.1); BDV-6: 91-F-7014 (EF693993.1), 94-F-7446/1 (EF693996.1), 06-F-0299/477 (EF694003.1), and 06-F-0299/369 (EF694001.1); BDV-7: 712/02 (AJ829444.1), TO/121/04 (AM900848.1), LA/82/04 (FM163383.1), LA/26/04 (FM163382.1), and LA/91/05 (FM163381.1); BDV-8: R4785/06 (MF102260), R9336/11 (MF102261), and Italy-58987 (KX573913); Turkey sheep pestiviruses: Burdur/05 (KM408491.1) and Aydin/04 (JX428945.1); Tunisian pestiviruses: SN2T (AF461996.1), SN3G (AY583306.1), and BM01/5 (AY453630.1); CSFV: Brescia (M31768.1), Alfort (X87939.1), and C-strain (Z46258.1); BVDV-1: Osloss (M96687.1) and NADL (M31182.1); BVDV-2: 890 (U18059.1) and C413 (AF002227.1); Giraffe-1: H138 (AF144617.2); Bungowannah pestivirus (EF100713.2); and Pronghorn (AY781152.3). Phylogeny was estimated using MEGA 5 [39] by the Neighbor-Joining (NJ) method and Kimura 2-parameter model of nucleotide substitution. Reliability of the trees was calculated using 1000 bootstrap replicates. 

### 4.6. GIS Analysis 

Geographic distribution of collected specimen, antibody prevalence, and virologically positive samples were represented via GIS (ESRI ARCGIS 10.3). 

## Figures and Tables

**Table 1:** Distribution of pestivirus in sheep farm in Sardinia: Number of flocks tested in this study and corresponding number of ewes per flock tested per Sardinian province, number of flocks tested in this study, percentage of seropositive flocks with 95% confidence interval (95% CI), number of flocks for each antibody inhibition percentage (AIP)-estimated seroprevalence class and PCR-positive flocks.

Province	Flocks	Total Number of Ewes	Tested Flocks (% of Total Flocks)	Ewes	ELISA Positive Flocks	% ELISA Positive Flocks (95% CI)	Flocks with Estimated Seroprevalence >30% 10–30%	PCR Positive Flocks
SS	3.460	928,003	291 (8.41)	90.268	17	5.84 (3.68–9.16)	7 10	2
NU	3.226	771,312	430 (13.33)	104.316	32	7.44 (5.32–10.32)	15 17	0
OR	2.118	470,560	189 (8.92)	60.239	21	11.11 (7.38–16.39)	11 10	1
SU	2.478	645,836	312 (12.59)	82.606	51	16.35 (12.66–20.85)	35 16	3
CA	253	58,370	64 (25.30)	17.409	24	37.5 (26.67–49.75)	18 6	3
TOT	11.535	2,874,081	1286 (11.15)	354.838	145	11.28 (9.66–13.12)	86 59	9

SS: Sassari, NU: Nuoro, OR: Oristano, SU: Sud Sardegna, CA: Cagliari, TOT: total number.

**Table 2:** Characteristics of the Sardinian border disease virus (BDV) strains sequenced in this study: strain, municipality/province, host, year, genotype, and GenBank accession number are reported.

Strain	Municipality/Province	Host	Year	Genotype	Accession Number
628	Barrali (SU)	Sheep	2015	BDV-7	MH733598
1018	Mandas (SU)	Sheep	2015	BDV-7	MH733599
1046	Ortacesus (SU)	Sheep	2015	BDV-7	MH733600
48550	Sedilo (OR)	Sheep	2014	BDV-7	MH733601
94863	Trinità d’Agultu (SS)	Sheep	2014	BDV-7	MH733602
66343	Siliqua (CA)	Sheep	2014	BDV-7	MH733603
49712	Perfugas (SS)	Sheep	2014	BDV-7	MH733604
80330	Las Plassas (CA)	Sheep	2014	BDV-7	MH733605
84294	Orroli (CA)	Sheep	2014	BDV-7	MH733606
